# Pulmonary laceration

**DOI:** 10.36416/1806-3756/e20240270

**Published:** 2024-11-16

**Authors:** Edson Marchiori, Bruno Hochhegger, Gláucia Zanetti

**Affiliations:** 1. Universidade Federal do Rio de Janeiro, Rio de Janeiro (RJ) Brasil.; 2. University of Florida, Gainesville (FL) USA.

**Figure 1:**
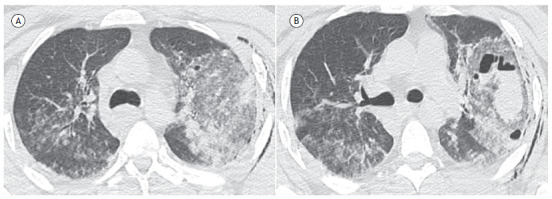
Axial CT scans with lung window settings showing extensive ground-glass opacities in the left lung (pulmonary contusion), interspersed with oval images with air and fluid content, forming air-fluid levels (pulmonary lacerations). Also note soft tissue emphysema and discrete ground-glass opacities on the right.

A 38-year-old man with no comorbidities was admitted to the emergency room after a motorcycle accident, complaining of chest pain, cough, and hemoptysis. A CT scan of the chest showed ground-glass opacities in the left lung, interspersed with oval formations with air and fluid content, forming air-fluid levels (Figure 1). A diagnosis of pulmonary laceration was made. The patient was treated conservatively, with no complications.

Pulmonary laceration (traumatic pneumatocele or traumatic pulmonary pseudocyst) is an uncommon injury associated with chest trauma that occurs as a consequence of traumatic rupture of the lung parenchyma. Due to the elastic nature of lung tissue, lacerations occur secondary to rapid compression and decompression of the parenchyma, forming cavities that fill with air or blood. Pulmonary lacerations are more commonly seen in children and young adults because of the greater flexibility of the chest wall in these age groups.^1^ ^-^ ^4^

The most common CT finding is a round or oval cystic mass with or without an air-fluid level. The lesion is usually surrounded by ground-glass opacities or consolidations resulting from pulmonary contusion. In the first 48 to 72 hours after trauma, pulmonary lacerations may be obscured on imaging by the underlying contusion. Once the contusion resolves, lacerations become apparent. They may be single or multiple, uniloculated or multiloculated, and rarely appear bilaterally. Associated CT findings may include pneumothorax, hemopneumothorax, rib fracture, or pneumomediastinum. Pulmonary lacerations usually resolve within 3-5 weeks. Treatment is primarily supportive, as the clinical course is usually benign. Serial imaging studies are helpful in demonstrating the gradual reduction in lesion size. Spontaneous resolution is expected within weeks to months.^1^ ^-^ ^4^

The diagnosis of pulmonary laceration is based on a history of chest trauma and imaging findings. Patients may be asymptomatic or present with cough, chest pain, dyspnea, hypoxemia, and hemoptysis. Hemoptysis may persist for up to 2 weeks. Because lesions may persist for weeks after trauma, correct diagnosis is critical to avoid confusing these alterations with cavitated lung lesions of other etiologies (abscess, tuberculosis, fungal diseases, malignancies, and others).^1^ ^-^ ^4^

Slow resolution, sometimes combined with a lack of details of the medical history of the patient, can lead to unnecessary invasive procedures due to suspicion of neoplasia or other diseases in some cases.
